# HCV and HIV Infection among Heroin Addicts in Methadone Maintenance Treatment (MMT) and Not in MMT in Changsha and Wuhan, China

**DOI:** 10.1371/journal.pone.0045632

**Published:** 2012-09-21

**Authors:** Xuyi Wang, Linxiang Tan, Yi Li, Yao Zhang, Dongyi Zhou, Tieqiao Liu, Wei Hao

**Affiliations:** 1 Mental Health Institute of the Second Xiangya Hospital, Central South University, Changsha, Hunan Province, China; 2 Mental Health Centre of Wuhan, Wuhan, Hubei Province, China; 3 The Psychiatry Hospital of Changsha, Changsha, Hunan Province, China; Yale School of Public Health, United States of America

## Abstract

**Objective:**

To compare HCV and HIV infection among heroin addicts in MMT and not in MMT in two large cities in central China.

**Methods:**

A total of 541 heroin addicts were recruited from MMT clinics and voluntary detoxification centers in Changsha and Wuhan, China. Structured questionnaires collected data on their socio-demographics, clinical status, risk behaviors, and their knowledge of HIV. Their HIV serostatus and Hepatitis C virus (HCV) serostatus were determined by testing antibodies in blood serum.

**Results:**

We observed a higher prevalence of HCV infection among MMT heroin addicts (82.3%) than that in the non-MMT group (50.6%). However, our findings indicated that the heroin addicts in MMT had less drug or sexual HIV/HCV risk behaviors and more knowledge about HIV than non-MMT addicts. The heroin addicts in MMT had a significantly higher percentage of individuals who always used condoms (44.9%) compared with patients in the non-MMT group (14.6%, p = 0.039), and they had more knowledge about HIV than non-MMT individuals (p<.001). The percentage of HIV-positive addicts in the MMT group (0.7%) and non-MMT group (0.8%) were almost same.

**Conclusion:**

Our study indicated that the rate of HCV infection among heroin addicts among MMT or non-MMT settings in central China is very high. The non-MMT heroin addicts have higher risk of becoming infected with HCV in the future, while at present they have lower rates of HCV infection than MMT heroin addicts. Although rates of HIV infection among MMT and non-MMT heroin addicts are low now, they are all at great risk of becoming infected with HIV in the future, especially for non-MMT heroin addicts. We should use the MMT sites as a platform to improve the control of HCV and HIV infection in heroin addicts.

## Introduction

Infection with HCV, which can develop into liver cirrhosis, liver failure, and hepatocellular carcinoma, causes heavy disease burden in the world [1]. Unlike in North America and Europe, the HCV pandemic has not been systematically studied and characterized in China. No population-based prevalence or incidence estimate is available [2]. The predominant sources of HCV infection in China are believed to be unsafe iatrogenic transmission and injection drug use [3].Like all the blood-borne viruses, HCV infections are efficiently transmitted through unsafe drug injection, and even more efficiently spread by this practice than is human immunodeficiency virus (HIV) [1], [4].

Illicit opiate use is considered to be a major social and public health problem in both western and eastern countries. Beginning in the 1990s, China experienced a rapid increase in illicit drug use: the number of registered addicts in 2005 was 1.16million [5], [6], while the actual number of drug addicts was estimated to have reached 3.5 million. At least 75–85% of registered drug addicts used heroin and 50–70% of heroin users were injection drug users (IDUs) [5], [6].

The literature on HCV infection among Chinese drug users and related issues in China has been limited. There is no study to comprehensively profile the HCV infection in drug addicts or to document geographical variation in HCV prevalence in China. A meta-analysis of HCV epidemiological studies in injection drug users indicated that the HCV prevalence rate ranged from 11.43% (Shannxi) to 90.77% (Hubei) among IDUs in China [2], [7]. Drug addiction and related problems, particularly the growing epidemics of HIV and HCV, are now among the greatest challenges facing the country. Therefore, developing effective harm reduction strategies to reduce the harm of heroin abuse in China is an urgent public health priority. In recent years, the Chinese government has implemented harm reduction strategies, which include MMT programs, needle–syringe programs (NSP), outreach, and increasing access to HIV testing in order to control the spread of HIV, HCV, and other sexually transmitted diseases among high-risk groups. MMT, which has shown efficacy for the treatment of opiate addiction and subsequent reduction of the harms associated with heroin abuse in many other countries, has been increasingly implemented in China. The MMT strategy was initiated in China in 2004. Since then it has expanded into a nationwide program encompassing more than 680 clinics covering 27 provinces and serving some 242, 000 heroin addicts by the end of 2009 [6], [8].

MMT is an effective intervention for the management of heroin addiction, reducing levels of heroin use and of risky behaviors (injecting and needle sharing).Therefore, MMT reduces the risk of HIV infection among opiate-dependent patients [9], [10]. But the effectiveness of MMT to control the spread of HCV infection among IDUs has been disputed [11], [12].Despite the widespread use of MMT for opioid addiction in China, there are still many addicts who have never enrolled in MMT [13]. Psychotherapy, such as cognitive behavioral therapy, plays an important role during the rehabilitation phase. Unfortunately, psychotherapy in China has been offered to only a small portion of patients at MMT clinics and rehabilitation centers. In addition to MMT, a variety of self-help groups have opened as rehabilitation programs throughout China, such as 12-Step Rehabilitation, Narcotics Anonymous (NA), and the Day-top Prevention and Recovery Program. However, these programs were only available in very limited areas in China, such as Beijing, Shanghai, and Yunnan province. So MMT was almost the only available rehabilitation program for heroin addicts in central China. If heroin addicts in central China did not enroll in MMT, which means that they did not accept the treatment of rehabilitation program. So the terms “non-MMT” and “MMT” were designed to distinguish addicts who entered rehabilitation treatment or not, but not compare MMT with detox patients. The MMT heroin addicts usually had already finished detoxification, whereas non-MMT heroin addicts may have been in detoxification or already completed detoxification. Previous studies of prevalence of HIV and HCV infection among opiate-dependent patients are divided between studies focusing on IDUs and studies focusing on non-IDUs [7]. At the time of this writing, however, there were few available reports regarding the difference in prevalence of HCV infection among heroin abusers in MMT or not in MMT. Risky injection practices, sexual risk behaviors, and poor knowledge about HIV/AIDS, STIs, and blood-borne viruses put many heroin addicts at high risk of infection with HIV and HCV. This study investigated HCV and HIV among heroin abusers in MMT or not in MMT (i.e., in detoxification centers).

The objectives of the study were (1) To document and compare the prevalence of HCV and HIV infection among heroin-dependent patients in MMT and not in MMT; (2) To compare the knowledge about HIV and risk behaviors among heroin-dependent patients in MMT and not in MMT. Our goal was to provide insight into factors affecting HCV and HIV infection among heroin-dependent patients receiving MMT or not and to aid in the development of strategies to prevent HCV and HIV infection among heroin abusers in central China.

## Results

Overall, 72.8% of the subjects tested positive for HCV, and there was a significant difference between the MMT and non-MMT groups: 83.2% of MMT addicts were HCV positive and 50.6% of non-MMT addicts were HCV positive. The prevalence of HIV infection in all heroin addicts was 0.74%, and there were no difference between the prevalence of HIV infection in MMT and in non-MMT subjects (0.7% of MMT and 0.8% of non-MMT subjects).

Subject characteristics are summarized in Table 1. There were no differences in gender, education, marital status, employment situation, age at first drug use, money for drug per day, and HIV infection between the MMT group and non-MMT group. The MMT group had a significantly older age (39.8±7.52 years) than the non-MMT group (36.1±7.43years, p = 0.000). The proportion of drug injection history in the MMT group (75.5%) was significantly higher than that (53.5%) in the non-MMT group. Data on the subjects' knowledge about HIV, as well as about injection and sexual risk behaviors are provided in Table 2. The heroin-dependent patients in the MMT group reported a significantly higher proportion of always using condoms (44.9%) than patients in the non-MMT group(14.6%, p = 0.039).Comparing the sharing of syringes in the past year and not using condoms, the heroin-dependent patients in the non-MMT group reported a higher proportion than patients in MMT, although not at a statistically significant. Fewer heroin-dependent subjects in the non-MMT group than patients in MMT knew that treatment for HIV is free (p = 0.000).

**Table 1:** Demographic and clinical characteristics of MMT subjects and non-MMT subjects.

	MMT	Non MMT	T or *X* ^2^-value	P
Population	294	247		
Age(Mean ± SD)	39.8±7.52	36.1±7.43	5.743	0.000
Male	224	199	1.507	0.220
Female	70	48		
Education				
<9 years	199	175	0.629	0.428
>9 years	95	72		
Employed				
No	83	84	2.099	0.147
Yes	211	163		
Marital status				
Married	159	134	0.002	0.969
Never married	97	93	1.278	0.258
Divorced	38	20	3.269	0.071
Age of onset drug abused	27.2±6.72	26.4±6.67	1.373	0.170
Methods of using heroin				
Injection	222	138	29.37	0.000
No injection	72	120		
Money for drug per day	357±208	355±215	0.124	0.901
HIV				
Positive	2	2	0.029	0.864
Negative	291	245		
Positive rate	0.7%	0.8%		
HCV				
Positive	242	125	64.81	0.000
Negative	52	122		

**Table 2:** Risk behaviors and knowledge about HIV among MMT and non-MMT subjects.

	MMT	Non MMT	T or *X* ^2^-value	P
Drug-related Risk	294	247		
Heroin injection history	222	138		
Share syringes in the past year	72	55	23.26	0.000
No share	150	83		
Age of first sex behavior	19.9±2.45	19.4±2.59		
Condom use in recent years				
Never	37	44	2.883	0.090
Always	132	36	0.319	0.039
Know where you can get HIV exam				
Yes	131	104	0.329	0.566
No	163	138		
Know Treat for HIV is free				
Yes	145	73	20.23	0.000
No	154	169		

Using logistic regression, we examined predictors of HCV serostatus (see Table 3). Only injection use, sharing syringes in the past year, and always using condoms were significant predictors of HCV-seropositive status. There was no significant predictor of HIV positive serostatus found in this study.

**Table 3:** Predictors of HCV Positive Serostatus.

P value		OR	95%CL	
Lower				Upper
Gender	0.817	0.94	0.55	1.59
Heroin injection	0.000*	9.60	6.00	15.43
Share syringes in the past year	0.000*	2.6	1.62	4.28
Age	0.155	1.0	0.99	1.07
Length of drug using	0.152	1.0	0.99	1.07
Condom use in recent years				
Never	0.107	1.5	0.91	2.53
Always	0.035*	0.5	0.28	0.96

## Discussion

All the subjects were enrolled from two large cities: Changsha, the capital city of Hunan province, and Wuhan, the capital city of Hubei province. Hunan and Hubei provinces are representative of central China in terms of their geographic location, population, and economy. To our knowledge, this is the first report comparing the rate of HCV infection between MMT and non-MMT heroin addicts in China. This study offers a unique examination of the rate of HCV infection and risk behaviors in heroin-dependent MMT patients as compared to addicts who are not enrolled in MMT.

Our findings confirm that the rate of the HCV infection is very high among opiate drug users in central China. The prevalence of HCV infection among heroin addicts was 72.8% (83.2% in the MMT group and 50.6% in the non-MMT group), which is consistent with earlier reports in other provinces in China [7] and worldwide [1], [14]. There are several reasons to explain why heroin addicts are a high-risk group for HCV infection. For IDUs, the main reason is the shared use of injecting equipment and drug solution, which contributes to HCV transmission [14]. Those who use heroin but have never injected may be at increased risk of becoming infected through sexual transmission compared with members of the non–drug-using population since they may be more likely to engage in sexual risk behaviors and to be exposed to infected drug users. In addition, opiate abusers have higher incidence of infectious diseases, including HIV and HCV, which may be directly related to the negative impact of opiate abuse on the host immune system [14]–[16].

The finding that the heroin addicts in MMT had higher prevalence of HCV infection than non- MMT heroin addicts is somewhat surprising since the MMT program is regarded as the most important harm-reduction program in China [8]. Public security and public health sectors thought methadone had dual roles as a means of HIV prevention as well as a method of drug control. Previous studies found that MMT has superior levels of retention in treatment, reduces and/or eliminates the use of heroin, reduces mortality and levels of crime associated with heroin use, and allows patients to improve their health and social productivity. In addition, enrollment in methadone maintenance has the potential to reduce the transmission of infectious diseases associated with heroin injection, such as hepatitis and HIV [10], [12], [14], [17]–[19]. We thought that our results were inconsistent with previous studies because the differences of demographic and clinical characteristics between MMT and non-MMT heroin addicts.

The heroin abusers in MMT (39.8±7.52 years) were older than non-MMT ones (36.1±7.43years), while the age of onset of drug abuse was similar for both groups; this means the heroin abusers in MMT had longer heroin abuse histories than non-MMT addicts. Compared with non-MMT heroin addicts, more heroin addicts in MMT injected heroin. We did explore predictive factors associated with risk of HCV infection in this study and found injection heroin, share syringes in the past year, and always using condoms were significant predictors of HCV infection. Our findings were partly consistent with previous studies that indicated that HCV infection can be positively correlated with injection drug use, and drug or sexual risk behaviors [14], [16], [20]–[22]. It is general consensus that the length of drug use was positively correlated with the HCV infection, although our study did not confirm it [14], [16], [20]–[22]. The differences in length of drug use and the greater injection drug history are plausible explanatory factors in the differences in prevalence of HCV infection between MMT and non-MMT heroin addicts. Our results contribute, from another perspective, to a growing body of literature reporting that injection drug use and longer drug use history are associated with high prevalence of HCV infection [7], [14], [20], [21]. In order for heroin addicts to understand the need to avoid such behaviors, we must increase their knowledge about HCV and reduce their risk behaviors.MMT programs are well situated to fill an important HCV education service gap among drug users by providing HCV education to their patients.

The finding that the heroin addicts in MMT are older than the addicts not in MMT mainly is attributable to the inclusion criteria of MMT in China. Only heroin addicts who have been registered by police and have at least two relapse experiences after detoxification can be enrolled MMT in China, while less-experienced heroin addicts usually avoid detection by police. So heroin addicts in MMT usually have longer duration of heroin use than addicts not in MMT.

The very high level of drug or sexual risk behaviors was revealed among the heroin addicts enrolled in this study, including injection of heroin, sharing of syringes, unsafe sexual behaviors, and so on. We believed those drug or sexual risk behaviors could explain why heroin addicts have very high proportions of HCV infection in this study. We did observe that HIV infection proportions among both MMT and non-MMT heroin users in this study were very low (0.7% in MMT and 0.8% in non-MMT). Considering that HIV and HCV share almost the same transmission routes and risk factors for infection, we suggest that the low rate of HIV infection is not because of absence of the risk behaviors for HIV but because the number of individuals who are HIV positive among heroin abusers is very low in Changsha and Wuhan, at least at present. In China, HIV prevalence has wide geographic variation: the rates of HIV infection among the heroin users were very high (the HIV prevalence was about 50% among IDUs) in the provinces that were close to the heroin trafficking routes, such as Yunnan, Guangxi, and Xinjiang provinces [7]. Changsha and Wuhan are close to Yunnan and Guangxi provinces, and population flow all over China has increased. These aspects of the present situation regarding sources of HIV infection in these two cities will be changing soon. There is a great public health risk from heroin addicts in central China putting themselves and others at risk of transmission of HIV (as well as other infectious diseases, including HCV).

Whereas we observed a higher prevalence of HCV infection among MMT heroin addicts, our findings indicated that the MMT group had a significantly higher rate of always using condoms (132/294) than subjects in the non-MMT group (36/247, p = 0.039). Comparing the rate of sharing their syringes in the past year *(P = 0.152)* and the rate of never using condoms *(P = 0.081)*, the rates among heroin users in the non-MMT group were higher than those in MMT, although the difference was not statistically significant. Fewer heroin users in the non-MMT group than subjects in MMT knew that treatment for HIV is free (p = 0.000). The heroin addicts in MMT reported engaging in less drug use or sexual risk behavior, potentially reducing the transmission of infectious diseases, and they had more knowledge about HIV than non-MMT addicts, which is consistent with previous findings that MMT can reduce risky behaviors [12], [19]. A meta-analysis of HCV epidemiological studies in injection drug users found a high risk of acquiring HCV infection rapidly after initiation of injection practices [23].These findings may illustrate that a majority of the heroin addicts in MMT may become HCV infected prior to seeking MMT. The higher level of risk behaviors among non-MMT heroin addicts than in MMT patients revealed by the present study is alarming, which may mean that more non-MMT heroin addicts will become infected with HCV and HIV in the future than MMT patients. The lack of knowledge about HIV and HCV may prevent heroin addicts from implementing measures that could reduce their risk of contracting HIV and HCV, thereby increasing the likelihood of transmission to others.

As mentioned above, international research has shown the efficacy of MMT for the treatment of opiate addiction and subsequent reduction in risk behaviors. Our study also confirmed it. The rapid development of the MMT program in China makes MMT the most feasible harm reduction strategy. The infrastructure of MMT sites provides an important opportunity for the provision of HCV/HIV education, prevention, and treatment. So we suggest that MMT is an appropriate setting for the identification of HCV/HIV infection and linkage to needed counseling and health care in China. We should enroll non-MMT heroin abusers into MMT as soon as possible, and we should improve the services to meet patient problems and needs.

However, there are still shortcomings inherent in MMT in China. Although as part of the routine intake procedure all patients admitted to MMT in China are tested for HCV, HIV, and other medical conditions, most clinics do not offer more comprehensive services such as counseling or referral for HCV/HIV prevention and treatment. Low coverage of the total opioid-using population and high client dropout rates are two very common challenges facing MMT of China. Further research is needed to better understand barriers and facilitators for successful implementation of the program. Such research could ultimately lead to lower rates of transmission of HCV/HIV among MMT patients.

There are several limitations in our study. First, we recruited a volunteer population from two MMT sites and two detoxification centers in the capital cities of Hunan and Hubei provinces. Since big cities can provide better health care for drug abusers than smaller cities in China, we cannot ensure that our estimate of HCV prevalence can be directly generalized to the entirety of the two provinces or other provinces. Second, All information, including heroin use and risk behaviors, was self-reported. Such information may be under-reported due to inclination of interviewees to give socially desirable responses. We cannot exclude the possibility of reporting bias among drug abusers, especially under-reporting of risk behaviors. Third, we did not track the change of prevalence of HCV and HIV infection in MMT or non-MMT heroin abusers, which should further illustrate the necessity of HCV prevention to heroin abusers.

Notwithstanding these limitations, the results indicate that the rate of HCV infection among heroin users in central China is a serious concern. The non-MMT heroin addicts have great risk of HCV infection in the future, even though they had lower rates of HCV infection than heroin addicts in MMT. Although rates of HIV infection among MMT and non-MMT heroin addicts are low now, they are at great risk of infection in the future, especially for non-MMT heroin addicts. We should use the MMT sites as a platform to increase earlier diagnosis and to improve treatment uptake for control of HIV and HCV infection. Future efforts need to focus on education and behavioral interventions to prevent HCV/HIV infection and transmission and to provide access to treatment in MMT in China.

## Methods

### Study Participants

A total of 541heroin-dependent volunteers were recruited from Changsha and Wuhan. These two large cities are the capital cities of Hunan and Hubei provinces, respectively, located in central China. The first group consisted of 294 opioid-dependent MMT patients who were recruited from MMT clinics in Changsha and Wuhan. The second group was a comparison group consisting of 247 non-MMT heroin-dependent patients who were recruited from voluntary detoxification centers in Changsha and Wuhan. These non-MMT individuals were hospitalized for treatment of withdrawal symptoms and had never been enrolled MMT before.

All these subjects met the Diagnostic and Statistical Manual of Mental Disorders, 4th edition (DSM-IV) criteria for lifetime heroin dependence determined from the Structured Clinical Interview (SCID). They were all over 18 years old. Study participants were asked to complete a series of questionnaires involving their socio-demographics and risk behaviors. They also provided consent to share their medical test results, including HIV and HCV testing.

### Ethics Statement and Informed Consent Procedures

Licensed psychiatrists conducted all clinical interviews. The Central South University Review Board approved all procedures used. Subjects were fully informed about the aim of the study. Written informed consent was given by all subjects. We protect the privacy and confidentiality of all of subjects. The individual in this manuscript has given written informed consent to publish these case details.

The investigators read the informed consent form to each of subject and made sure each subjects understood all the details about context of informed consent form. We explained the potential risks/discomforts and anticipated benefits to the subjects during this study. We emphasized that the subject's participation was voluntary. All of them were free to decide whether or not to participate and to withdraw their consent at any time. Prospective participants were assured that their decision would not cause any penalty or disadvantage to their future care in MMT clinics or in voluntary detoxification centers. After completing the informed consent, each subject received a medical exam and tests, then each of them spent one hour answering the questionnaires.

The informed consent form in this study was written in Chinese. The context of this form included the aim of this study, the procedure of this study, the potential risks and anticipated benefits to the subjects during this study, and confidentiality for the information of subjects. The sample copy of an informed consent form (Chinese Version) of this study can be found in File S1.

### Instruments/measures

A structured questionnaire was used to record the information of all the participants. In addition to demographics and drug use history, the questionnaire included items about risk behaviors (such as drug injection, needle sharing, and unprotected sex behaviors) and the knowledge about blood-borne and sexually transmitted disease.

### Statistical analyses

Two-sample t-test and Chi-square test was used to compare the differences between MMT and non-MMT participants. The level of statistical significance was set at p<0.05. Analysis was performed using SPSS 13.0.

We used logistic regression to identify predictors of HCV and HIV serostatus. In preliminary analyses, we included demographics(gender, age,) drug risk behaviors (injection use, sharing syringes, and length of drug use history) and sexual risk behaviors (condom use) in the logistic regression predicting HCV and HIV serostatus. The significant P-value is set at 0.05or less.

## Supporting Information

File S1 Informed consent form.
(DOC)
