# Supplementary material for: HCV and HIV Infection among Heroin Addicts in Methadone Maintenance Treatment (MMT) and Not in MMT in Changsha and Wuhan, China
Source: PLoS One. 2012 Sep 21;7(9):e45632. doi: 10.1371/journal.pone.0045632 (PMC3448629; doi:10.1371/journal.pone.0045632)
Supplement: File S1 — Informed consent form. (DOC) [file pone.0045632.s001.doc]

**知情同意书**

**研究编号：|__|__|-|__|__|__|-|__|-|__|**

研究题目：我国吸毒人群中艾滋病的流行规律、疫情预测和评估、网络监测和干预措施

项目负责人： 北京大学中国药物依赖性研究所 陆林、刘志民 教授

云南省药物依赖防治研究所 李建华 教授

中南大学湘雅二院精神卫生研究所 郝伟 教授

上海市精神卫生中心 赵敏 教授

资助单位：中华人民共和国科学技术部

引言

现在邀请您参加一项研究，针对严重影响人民健康和生活的传染性疾病，它们是艾滋病和丙型肝炎。这项研究的目的是要了解成瘾物质使用人群（海洛因依赖者和新型毒品使用者）中有多少人感染了这二种疾病。这些信息将用来帮助阻止这些疾病的传播。

我们邀请您参加这项调查，是因为您是当地登记在册的成瘾药物使用者（包括海洛因滥用者或新型毒品使用者），年龄18-64岁。在本地区所有登记在册的成瘾药物使用者都可以自愿参加这项调查。

这是一份知情同意书，它包含本研究的有关情况，我们研究人员将向您作介绍，在这个过程中，您可以随时提问。如果您同意参加这项研究，请在下面签名或按手印，您将保留这份同意书的副本。如果您不同意参加，您的一切利益不会受到任何影响；您可以在本研究的任何时候退出，您的一切利益也不会受到任何影响。如果您决定从本研究中退出，您不必再做任何其他的事。如果您不能遵照研究所规定的程序，或如果该研究被资助方中国政府或管理机构、或伦理审查委员会（伦理审查委员会是一个监视研究对象安全和权利的机构）所取消，研究人员可能在没有获得您许可的情况下提前终止您参加本研究。

研究过程解释

我们的研究将在云南省、湖南省、上海市、北京市或陕西省抽取的美沙酮维持治疗门诊、社区戒毒社区康复场所或强制隔离戒毒所进行。调查对象由三部分构成：一部分为在当地新入组（入组1-6个月）美沙酮维持治疗的海洛因滥用者都可以自愿参加本次“阿片类物质使用情况及HIV高危行为调查问卷”的调查，同时进行艾滋病等疾病的筛查，并接受一年两次的随访（在2009年后半年和2010年将被重复2次）；第二部分为社区内的非美沙酮维持治疗的海洛因滥用者也可自愿参加本次“阿片类物质使用情况及HIV高危行为调查问卷”的调查，同时进行艾滋病等疾病的筛查；第三部分为目标地区的强制隔离戒毒所新如所的“新型毒品”使用人群都可以参加本次““新型毒品”使用情况及HIV高危行为调查问卷”调查，同时进行艾滋病等疾病的筛查。签署这份知情同意书，表示您同意自愿参加这一轮调查。

完成本研究大约需要40分钟。如果您同意参加，我们的调查员将单独地问您一些问题，包括您的一般情况、药物使用、性生活、对艾滋病的知识和态度等情况；之后，调查员会了解您的个人情况，和您讨论有关艾滋病和丙型肝炎的发病、传播及预防常识，并分析检测的利弊；如果您近2个月内没有做过艾滋病和丙肝检测，我们将从您的前臂抽5毫升静脉血用于检测艾滋病和丙肝指标。

检测后大约四个星期后，调查人员将会把您的检测结果反馈给您，并根据您的检测结果针对性的给您讲解药物滥用知识和HIV感染的高危行为及预防方法等咨询。如果您知道了自己的感染状况和接受了检测后咨询，就可以更好地照料自己，及防止把疾病传播给他人。并且我们的医务人员将给您讲解药物使用相关HIV感染的高危行为及预防方法，您参加该研究是完全自愿的，你可以在中途任何时候改变注意，不会受到任何惩罚。

保密

您的所有个人记录包括问卷和化验结果都是保密的。我们不会把结果告诉别人，包括您的家人和朋友。而且，问卷及您的化验结果都用数字编号，因此本研究的工作人员和审查人员（本研究资助单位和北京伦理审查委员会）只能看到记录上的数字编号，而不能看到您的姓名，以后发表的研究结果也不包括您的个人信息。

如果您感染了艾滋病，我们建议您自己把结果告诉给您的配偶，采取必要的防护措施以防止疾病的传播。按照法律规定，我们要把您的姓名和地址告诉当地疾病预防控制中心，当地卫生部门的工作人员会和您联系。

风险和不适

有些调查问题可能让人难以回答，您不必回答让您感到不舒服的问题。采血过程不会把艾滋病和其他疾病传染给您，因为给您采血用的注射器和针头都是新的和经过消毒的。抽取的少量血液不会影响您的健康。采血可能会引起局部轻微疼痛、淤青、红肿或感染，极少数人可能感到头晕。您等待化验结果期间可能会感到焦虑。如果您发现您感染了这两种疾病，您可能感到不安和害怕。如果别人发现您感染了，可能对您另眼相待。但我们受过专门训练的咨询员将帮助您处理这些感受和问题。

利益

您参加本研究的好处是：如果您知道感染了艾滋病或丙肝，您就可以尽早采取措施，保护家人免受感染。如果您感染了艾滋病，您会得到免费的艾滋病抗病毒治疗。目前中国尚缺乏丙肝的治疗方法，所以丙肝患者将不被治疗。

对参加研究的补偿

您参加本研究不需要花钱，所有化验和咨询都是免费的。

问 题

如果您对本研究有任何疑问，或万一遇到因研究而造成的伤害时，您可以拨打电话010-82802459与北京大学中国药物依赖性研究所所长陆林教授，或拨打电话010-82802460与北京大学中国药物依赖性研究所副所长刘志民教授，或拨打电话0871-8055507与云南省药物依赖防治研究所李建华副所长，或拨打电话021-54252689与上海市精神卫生中心赵敏副院长或拨打电话0731-5292156 与中南大学湘雅二院精神卫生研究所副所长郝伟联系。

签名

在下面签名表示您自愿参加：

___________________ ________________ _____年___月___日

参加者姓名（书写工整） 参加者签名或按手印 日 期

___________________ ________________ _____年___月___日

调查员姓名（书写工整） 调查员签名 日 期

___________________ ________________ _____年___月___日

证人姓名（书写工整） 证人签名 日 期
